# Thrombospondin 1 Promotes Cytoskeleton Remodeling, Dedifferentiation, and Pulmonary Metastasis through ITGA1 and ITGA6 in Osteosarcoma

**DOI:** 10.7150/ijbs.93678

**Published:** 2025-02-18

**Authors:** Enjie Xu, Zhen Huang, Kunpeng Zhu, Jianping Hu, Xiaolong Ma, Yongjie Wang, Jiazhuang Zhu, Chunlin Zhang

**Affiliations:** 1 Department of Orthopedic Surgery, Shanghai Tenth People's Hospital, School of Medicine, Tongji University, Shanghai, 200072, PR China.; 2 Institute of Bone Tumor Affiliated to Tongji University School of Medicine, Shanghai, 200072, PR China.

**Keywords:** osteosarcoma, dedifferentiation, metastasis, cytoskeleton remodeling, cell adhesion, THBS1

## Abstract

Dedifferentiation of osteosarcoma cells leads to poor prognosis. We plan to identify the key molecules that are involved in cell dedifferentiation and explore how they promote the pulmonary metastasis of osteosarcoma cells. We performed a sphere formation assay and confirmed that the spheroid cells could be redifferentiated into osteoblasts, adipocytes, and chondrocytes in specific medium, and the stem cell-like markers Stro-1 and CD117 were detected on the cell surface, which indicated that the spheroid cells were dedifferentiated cells. Thrombospondin 1 (THBS1) and ITGAs were identified as the key molecules in dedifferentiation through mRNA-seq and analysis, and osteosarcoma patients with higher THBS1 expression had a worse prognosis than those with lower THBS1 expression. THBS1 promotes the accumulation of ITGA1 and ITGA6 on the cell membrane in the early phase of dedifferentiation, thereby increasing the phosphorylation of FAK, RasGRF1, and MLC2 in the cytoplasm and promoting cytoskeleton remodeling. Our results suggest that THBS1 promotes cell dedifferentiation and pulmonary metastasis by promoting cytoskeletal remodeling and that ITGA1 and ITGA6 play important roles in mediating extracellular to intracellular signals; this mediating effect takes place mainly in the early phase of dedifferentiation.

## Introduction

Osteosarcoma is most commonly diagnosed in children and young adults, and the long-term survival rates remain low due to pulmonary metastasis^1^. The vast majority of patients already show micrometastasis at the time of diagnosis^2^. The overall survival rate for osteosarcoma is approximately 60-70% over a five-year period^3^. However, metastatic osteosarcoma presents significant treatment challenges, leading to overall survival rates of less than 20%^4^, ^5^. Numerous studies have demonstrated that proteins and signaling pathways—such as ezrin, TGF-β, MMPs, RUNX2, Notch, Wnt/β-catenin, and PI3K/AKT—are critical factors in the metastasis of osteosarcoma^6^. Some of these metastasis related molecules are also associated with the stemness of osteosarcoma cells. TGF-β appears to enhance stemness. Ma *et al.* demonstrated that knocking down TGF-β reduced the expression of stem cell markers in osteosarcoma cells^7^. Additionally, there appears to be a connection between Notch signaling and stem cells^8^, ^9^. Many miRNAs associated with osteosarcoma metastasis can also target stem cell markers, thereby influencing stem cell activity either positively or negatively. One notable example is miR-487b-3p^6^.

Recent studies in several types of human cancers showed that tumor cells are heterogeneous, and dedifferentiated cells are a subpopulation of cancer cells with stem cell-like self-renewal ability and multipotency^10^. The process by which mature, differentiated cells revert to a less differentiated, stem cell-like state is referred to as dedifferentiation. Dedifferentiated cells are radioresistant/chemoresistant and are associated with distant metastasis and poor prognosis^11^-^16^. Dedifferentiation occurs in osteosarcoma cells and is associated with tumor malignancy, including tumor metastasis. Zhang *et al.* demonstrated that stem cells and mature differentiated cells exist in a dynamic equilibrium within the osteosarcoma cell population and that cancer stem cells may develop de novo from differentiated cancer cells^17^. Stemness factors such as Sox2, Oct4, and Nanog are found in pathological specimens of osteosarcoma patients^18^. The dedifferentiation of osteosarcoma was proven to account for tumor growth and metastasis^19^, ^20^. CD117 and Stro-1, as markers for MSCs, are also double-positively expressed in some human and mouse osteosarcoma cells, which exhibit stronger multilineage differentiation potential, invasion, and metastasis ability^19^. CD133, a marker of dedifferentiation, is closely related to pulmonary metastasis and poor prognosis in osteosarcoma patients^21^. Lamhamedi-Cherradi *et al.* identified a subset of osteosarcoma cells termed derived circulating tumor cells (dCTCs) that showed less differentiation and highly expressed epithelial-to-mesenchymal transition-associated transcription factors (EMT-TFs)^22^.

Therefore, exploring how osteosarcoma cells dedifferentiate and finding solutions to inhibit dedifferentiation is necessary to help clinicians inhibit tumor pulmonary metastasis in patients with osteosarcoma. Zhang *et al.* found that blocking of TGF-β1 resulted in decrease of the dedifferentiation of osteosarcoma cells^17^. Additionally, several researchers have observed that the upregulation of miR-34a expression plays a crucial role in the dedifferentiation of osteosarcoma^23^. This finding suggests that miR-34a may represent a promising therapeutic target for preventing the dedifferentiation of osteosarcoma cells.

However, there are currently no studies that clearly illustrate how osteosarcoma cells dedifferentiate and detail which molecules could serve as targets to block the dedifferentiation of osteosarcoma cells. In this study, we aim to isolate dedifferentiated osteosarcoma cells and conduct mRNA sequencing analysis to identify key molecules involved in their dedifferentiation. We will explore how these molecules promote both dedifferentiation and lung metastasis of osteosarcoma cells. This research will serve as a foundation for the future development of drugs aimed at inhibiting dedifferentiation and lung metastasis in osteosarcoma.

## Materials and Methods

### Cell culture

DMEM (HyClone, Utah, USA) medium contained 10% fetal bovine serum (FBS, Gibco, NY, USA), penicillin (100 U/ L, Invitrogen, NY, USA), and streptomycin (100 mg/L, Invitrogen, NY, USA). McCoy's 5a (Invitrogen, NY, USA) medium contained 15% FBS, penicillin (100 U/L), and streptomycin (100 mg/L). DMEM/ F12 (HyClone, Utah, USA) medium contained 10% FBS, penicillin (100 U/L), and streptomycin (100 mg/L). U-2 OS cells were cultured in McCoy's 5a medium, MG-63 and 143B cells were cultured in DMEM, primary osteosarcoma cells were cultured in DMEM/F12, and all cells were placed in a humidified atmosphere with 5% CO_2_ at 37°C.

### *In vitro* dedifferentiation and redifferentiation

We employed a sphere formation assay to obtain dedifferentiated cells. The abovementioned osteosarcoma cells were cultured in 100 mm dishes until the cell confluence is approximately 80%-90%. The cells were washed with warm PBS, 1 mL of 0.05% trypsin-EDTA was added, and the cells were incubated at 37°C for 3 minutes. The mixture was then transferred to a 15 mL centrifuge tube, the cells were centrifuged at 200 × g for 5 minutes, and the cells were resuspended in 1 mL of serum-free medium. Matrigel (BD Biosciences, CA, USA) and the cell suspension were mixed at a ratio of 1:1, and the mixture was spread into the center of a 24-well plate culture dish at 5,000 cells per well. The plate was incubated at 37°C for 60 minutes until the Matrigel was cured. After solidification, 500 μL of warm serum-free medium containing ITS-X (BasalMedia Technologies, Shanghai, China) was added to each culture well, and the warm medium was replenished every 3 days.

We also performed redifferentiation experiments of spheroid cells according to the manufacturer's instructions for osteogenic differentiation medium, chondrogenic differentiation medium, and adipogenic differentiation medium (Cyagen Biosciences, Jiangsu, China).

### mRNA-seq and the identification of key molecules

High-throughput mRNA sequencing was conducted by OE Biotech (Shanghai, China). In brief, total RNA of the sarcospheres and residual cells was extracted, and magnetic beads with oligo (dT) were used to enrich eukaryotic mRNA. Then, cDNA was synthesized, and PCR amplification was performed. After quality inspection with an Agilent 2100 Bioanalyzer, sequencing was performed using an Illumina HiSeq 4000. Differentially expressed genes (DEGs) were identified using the DESeq package, and then the DEGs were used for Gene Ontology (GO) and Kyoto Encyclopedia of Genes and Genomes (KEGG) enrichment analysis. For protein-protein interaction (PPI) analysis, the proteins corresponding to all identified DEGs were submitted to the STRING database for to evaluate protein interactions. Then, the cytoNCA and MCODE plugins of Cytoscape software were used to analyze and cluster networks. We ranked all proteins based on their betweenness, and the size of each node in the networks represented the betweenness value of the corresponding protein. The settings for the MCODE plugin to explore key molecules were as follows: degree cutoff = 10, node score cutoff = 0.2, k-core = 2, and max.depth = 100^24^, ^25^.

### Knocking down or overexpression

Recombinant lentiviruses designed for the knockdown or overexpression of THBS1, as well as the knockdown of ITGA1 or ITGA6, along with a control blank lentivirus, were procured from Shanghai GeneChem Co.,Ltd. (Shanghai, China). These lentiviruses were subsequently used to transfect osteosarcoma cells. Initial preliminary experiments were conducted to determine the optimal multiplicity of infection (MOI) for viral infections, which guided our formal experimental protocols. To increase the proportion of infected cells, appropriate concentrations of puromycin or neomycin were incorporated into the culture medium, resulting in the selective enrichment of infected cells after a 3-day infection period. mRNA and protein extraction were performed once the cells reached 70-80% confluence. The effectiveness of the knockdown or overexpression was validated through RT-qPCR and Western blotting analyses.

### Immunocytochemistry (ICC)

The formed spheres or cells were fixed with 4% paraformaldehyde for 30 minutes, permeabilized with 0.1% Triton X-100 in PBS for 5 minutes, and blocked with blocking buffer (0.1% bovine serum albumin (BSA), 0.2% Triton X-100, and 10% normal goat serum (NGS) in PBS) for 2 h. Spheres/cells were incubated overnight with primary antibody at 4 ℃. The primary antibodies used were as follows: anti-Stro-1 (1:100, ab214086, Abcam), anti-CD117 (1:100, ab283653, Abcam), anti-THBS1 (1:100, ab267388, Abcam), anti-ITGA1 (1:50, sc-271034, Santa Cruz), anti-ITGA6 (1:50, sc-374057, Santa Cruz), and anti-ITGA10 (1:100, PA5-100840, ThermoFisher). The primary antibody was removed after incubation, the spheres/cells were gently washed with PBS, and then the spheres/cells were incubated with the secondary antibody (all secondary antibodies from Beyotime Biotechnology, Shanghai, China). For F-actin staining, cells were incubated with Alexa Fluor 647 phalloidin (1:20, CST) for 15 minutes at room temperature. Afterward, spheres/cells were mounted with DAPI (Sigma Aldrich) after being washed with PBS. Spheres/cells were observed and photographed under a confocal microscope (LSM900, Carl Zeiss, Germany).

### Immunohistochemistry (IHC)

With institutional review board approval from Shanghai Tenth People's Hospital, human osteosarcoma specimens were acquired from patients who underwent surgery. This study was performed in accordance with the Declaration of Helsinki. Osteosarcoma specimens and mouse lung tissues were fixed in 4% paraformaldehyde, embedded in paraffin, and subsequently prepared for hematoxylin and eosin (H&E) staining as well as IHC staining. We performed IHC staining using an IHC kit according to the manufacturer's instructions (Yeasen Biotechnology, Shanghai, China). The primary antibodies were utilized as follows: anti-THBS1 (1:5000, ab267388, Abcam), anti-Stro-1 (1:500, 14-6688-82, ThermoFisher), anti-CD117 (1:100, ab283653, Abcam), anti-phospho-MLC2 (1:50, AF3829, Affinity Biosciences).

### Western blotting and Co-Immunoprecipitation (Co-IP)

Cells from each group were collected, and membrane/membrane-associated proteins, along with cytosolic proteins, were isolated using a Mem-PER Plus Membrane Protein Extraction kit (Pierce, IL, USA). The protein concentration was determined by a BCA Protein Assay kit (Pierce) and equal amounts of cell extracts were run on SDS PAGE gels. Separated protein bands were transferred onto polyvinylidene fluoride (PVDF, Millipore, MA, USA) membranes and blocked in 5% BSA (Sigma-Aldrich, St. Louis, MO, USA). Membranes were incubated overnight at 4°C with the appropriate primary antibodies. The following antibodies were utilized: anti-THBS1 (1:1000, ab267388, Abcam), anti-ITGA1 (1:100, sc-271034, Santa Cruz), anti-ITGA6 (1:100, sc-374057, Santa Cruz), anti-ITGA10 (1:500, PA5-100840, ThermoFisher), anti-Na^+^/K^+^-ATPase (1:500, ab58475, Abcam), anti-FAK (1:500, A11131, ABclonal), anti-phospho-FAK (1:100, AP0302, ABclonal), anti-RasGRF1 (1:1000, DF8026, Affinity Biosciences), anti-phospho-RasGRF1 (1:1000, 82309-1-RR, Proteintech), anti-MLC2 (1:1000, 8505, CST), anti-phospho-MLC2 (1:1000, 3671, CST), anti-GAPDH (1:1000, AC001, ABclonal). We chose a loading control according to Abcam's recommendation. The relative protein level in different groups was normalized to Na^+^/K^+^-ATPase or GAPDH concentration. Membranes were then incubated with secondary antibodies (ABclonal) for 1 h at room temperature. The immunoreactive bands were visualized using BeyoECL Plus (Beyotime Biotechnology, Shanghai, China).

RhoA-GTPase activity was evaluated according to the manufacturer's instructions using the RhoA Pull-Down Activation Assay Biochem Kit (Cytoskeleton, USA). Subsequently, the protein samples were analyzed through Western blotting, as previously described.

Co-IP was applied using the Immunoprecipitation Kit with Protein A+G Agarose Gel according to the manufacturer's instructions (Beyotime Biotechnology, Shanghai, China). Immunoprecipitates were subjected to Western blotting analysis as described above.

### Wound-healing assay

Osteosarcoma cells from each group were cultured in 6-well culture plates and grown to 90% confluence. Wounds were created using a 1000 μL micropipette tip. The cells were then maintained in a serum-free medium. The migration of cells toward the wound was monitored and the wounds were photographed. The calculated cell migration rate = (0 h width - 12 h or 24 h width)/0 h width × 100%.

### Transwell migration and invasion assays

The migration and invasion abilities of cells were determined in a 6.5 mm transwell (Corning, NY, USA). For the invasion assay, the filter of the top chamber was coated with 50 μL diluted Matrigel. Then, 5×10^4^ osteosarcoma cells were suspended in serum-free medium and plated on the top chambers, and the lower chambers were filled with 500 μL medium containing FBS. The cells were cultured for 24 h, and the invading cells were fixed with 4% paraformaldehyde for 30 minutes and then stained with a crystal violet staining solution (Sigma Aldrich). Then, the non-invading cells were removed, and the number of invading cells was counted and analyzed by Image J software (NIH Image, MD, USA).

### Animal model and treatment

All experimental animal procedures were performed according to the protocols approved by the Animal Research Ethics Committee of the Shanghai Tenth People's Hospital. For the tail vein pulmonary metastasis model, 1×10^6^ 143B cells of each group were injected through the tail vein into 6-week-old female nude mice. For the orthotopic lung metastasis model, 2×10^6^ 143B cells from each group were injected into the proximal tibia of 6-week-old female nude mice. At 6 weeks after injection of cells, the mice were then sacrificed, and the harvested lungs were fixed in formaldehyde for histopathology analysis.

### Statistical analysis

The results were expressed as the mean ± standard deviation (SD). Differences between means were analyzed using a two-tailed t-test for comparisons between two groups and ANOVA for comparisons involving more than two groups. SPSS 20.0 (IBM, USA), and GraphPad Prism 9 (San Diego, USA) were used for all statistical analyses. Kaplan‒Meier survival curves were used to assess overall survival in osteosarcoma patients, and the results were then evaluated using the log-rank test. Statistically significant results are labeled as follows in all figures: *, P < 0.05; **, P < 0.01; and ***, P < 0.001.

## Results

### THBS1 and ITGAs are key molecules in the dedifferentiation of osteosarcoma cells

**Figure 1:**
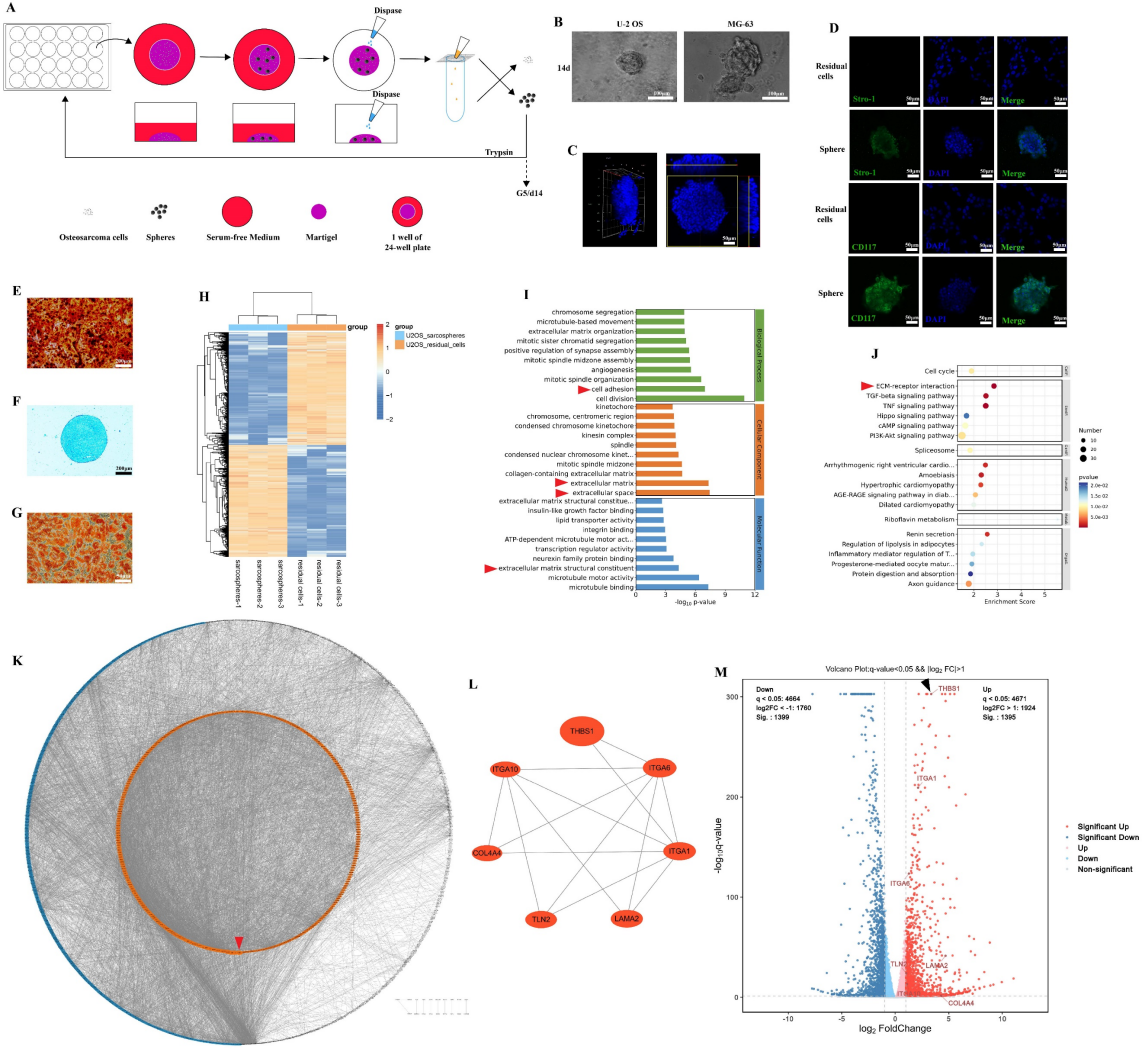
** Identification of the key molecules involved in the dedifferentiation of osteosarcoma cells.** (A) Scheme of the experimental procedure of sphere formation assay. (B) Sarcospheres of U-2 OS and MG-63 cells are shown. (C) The three-dimensional shape of a sphere is shown. (D) Immunocytochemistry was performed to determine Stro-1 (green) and CD117 (green) expression in residual cells and sarcospheres. Residual cells and sarcospheres were counterstained with DAPI (blue). (E-G) Osteogenic, chondrogenic and adipogenic experiments were performed. (H) Hierarchical clustering of the differentially expressed genes between formed spheres and residual cells was performed. Red indicates the upregulated genes and blue indicates downregulated genes. Each column represents a sample, and each row represents a differentially expressed gene. (I) GO functional enrichment analysis for differentially expressed genes was performed. (J) KEGG pathway enrichment analysis for differentially expressed genes was performed. (K) A PPI network among differentially expressed genes was constructed. The red arrow indicates THBS1. (L) The relationship among the seven key molecules at the protein level is shown. (M) A volcano plot was constructed with the cutoff criteria q < 0.05 and |logFC > 1. Red indicates upregulated genes, and blue indicates downregulated genes. Each circle represents a gene, and the identified key molecules are marked with different colors. The black arrow indicates THBS1.

Previous studies confirmed the presence of osteosarcoma stem cells by the sphere formation assay or cell sorting according to cell surface markers (CD117^+^/Stro-1^+^)^19^, ^26^. Osteosarcoma sarcospheres were formed in Matrigel-constructed 3D medium in 24-well plates (Figure 1A). After repeated cultivation, we observed that osteosarcoma cells formed spheres in Matrigel (Figure 1B-C). The spheroid cells were enriched in the expression of CD117 and Stro-1 when compared to the residual cells (Figure 1D). These spheroid cells could also redifferentiate into osteoblasts, chondrocytes, and adipocytes (Figure 1E-G). The results suggest that osteosarcoma cells underwent dedifferentiation during the process of sphere formation. Later, mRNA-seq was employed to explore the DEGs between residual cells and sarcospheres, and identify the key molecules in the dedifferentiation of osteosarcoma cells. In total, 2794 genes had significant differences in expression in sarcospheres compared to residual cells (p < 0.05 and |logFC| > 1); 1395 genes had markedly upregulated expression, and 1399 genes had downregulated expression. Hierarchical clustering clearly showed the landscape of genomic differences between sarcospheres and residual cells (Figure 1H). Functional annotations of GO enrichment indicated that these genes were significantly associated with cell adhesion, extracellular matrix, extracellular space, and extracellular matrix structural constituent (Figure 1I, red arrows). KEGG enrichment analysis results demonstrated that these genes were also related to ECM-receptor interaction (Figure 1J, red arrow). Then, we constructed a PPI network with the proteins corresponding to the DEGs and ranked all proteins based on their betweenness. The proteins arranged in the orange inner ring were relatively closely related to dedifferentiation, among which THBS1 had the strongest correlation (Figure 1K, red arrow). We determined a subnetwork module with significant prognostic values; of the molecules in the subnetwork, THBS1, ITGA1, ITGA6, ITGA10, COL4A4, TLN2, and LAMA2 were selected as key molecules based on their high degree of connectivity (Figure 1L). Among them, direct interactions had been shown between THBS1 and ITGA1, and between THBS1 and ITGA6 (Figure 1L). Furthermore, THBS1 is the most differentially expressed DEGs of these key molecules in dedifferentiation (Figure 1M, black arrow).

### THBS1 leads to worse prognosis and promotes dedifferentiation via ITGA1 and ITGA6

**Figure 2:**
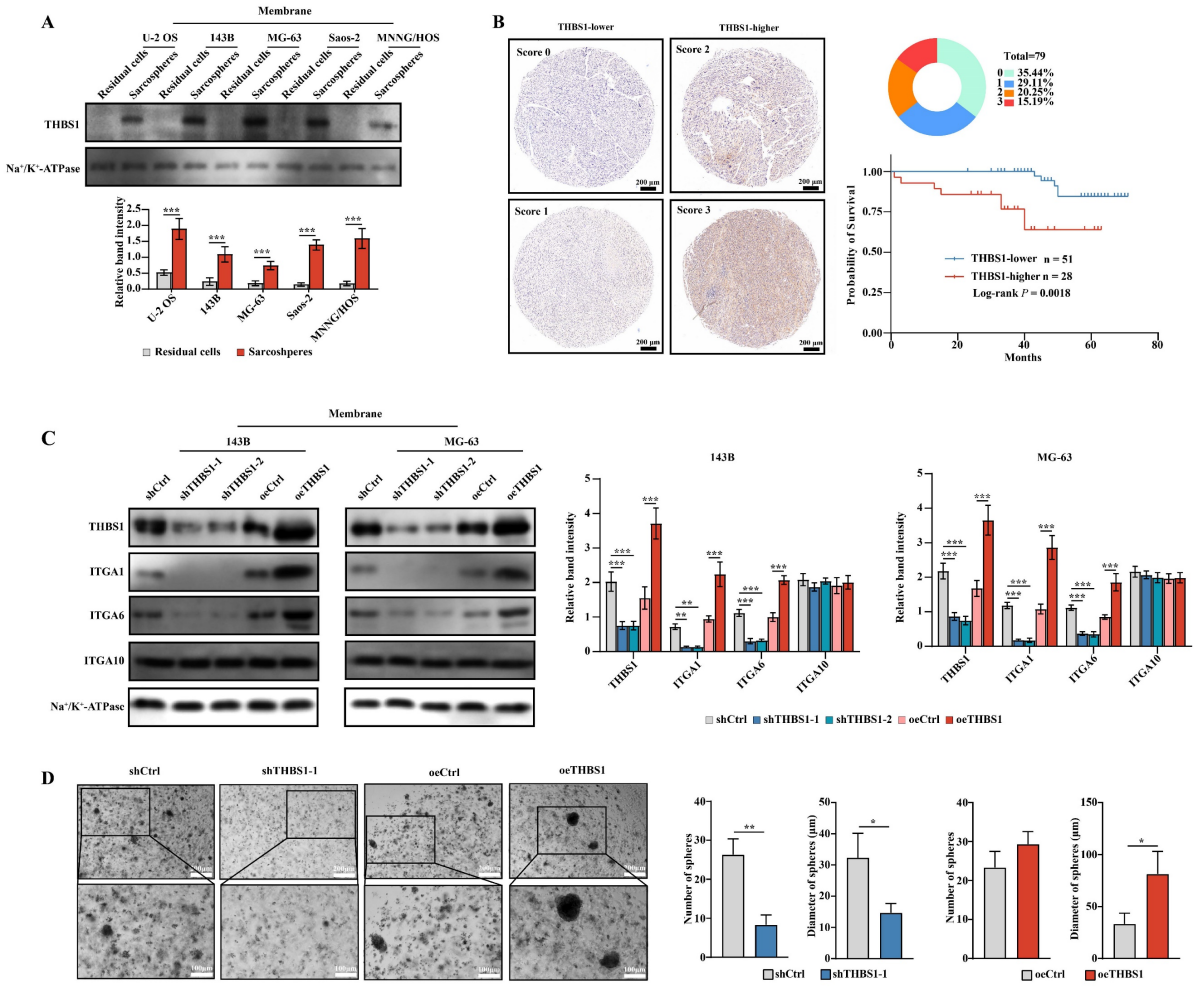
** THBS1 leads to worse prognosis and promotes dedifferentiation via ITGA1 and ITGA6.** (A) The expression of THBS1 between formed spheres and residual cells in different osteosarcoma cell lines was determined. (B) Representative IHC staining images of THBS1 in osteosarcoma specimens are shown and the Kaplan-Meier plot of overall survival based on THBS1 expression in osteosarcoma patients is shown. (C) The expression of THBS1, ITGA1, ITGA6 and ITGA10 in THBS1 knockdown and overexpression cells is shown. (D) The sphere formation ability of THBS1 knockdown and THBS1 overexpression cells was evaluated. ***P < 0.001, **P < 0.01, *P < 0.05.

The above results indicated that THBS1 and three ITGAs were key molecules in the dedifferentiation of osteosarcoma cells. We further employed Western blotting to confirm the above results in protein level, and found that in U-2 OS, 143B, MG-63, Saos-2 and MNNG/HOS cells, THBS1 was more highly expressed in sarcospheres relative to that in residual cells (Figure 2A). Then, we performed IHC on 79 human osteosarcoma specimens and observed that patients with higher expression of THBS1 had a worse prognosis (Figure 2B, log-rank *P* = 0.0018). Lentiviral transfection was employed to knockdown or overexpress THBS1 in the 143B and MG-63 osteosarcoma cell lines. The Western blotting results indicated that the expression of ITGA1 and ITGA6 changed after knocking down or overexpressing of THBS1, but no similar significant changes occurred in terms of ITGA10 expression (Figure 2C). We also attempted to confirm whether knockdown or overexpression of THBS1 would affect osteosarcoma cell dedifferentiation, and a sphere formation assay showed that knocking down THBS1 significantly reduced the number and diameter of sarcospheres, while overexpression of THBS1 increased the diameter of sarcospheres, indicating that THBS1 affects the dedifferentiation of osteosarcoma cells (Figure 2D).

### THBS1 promotes migration and invasion of osteosarcoma cells through cytoskeleton remodeling

**Figure 3:**
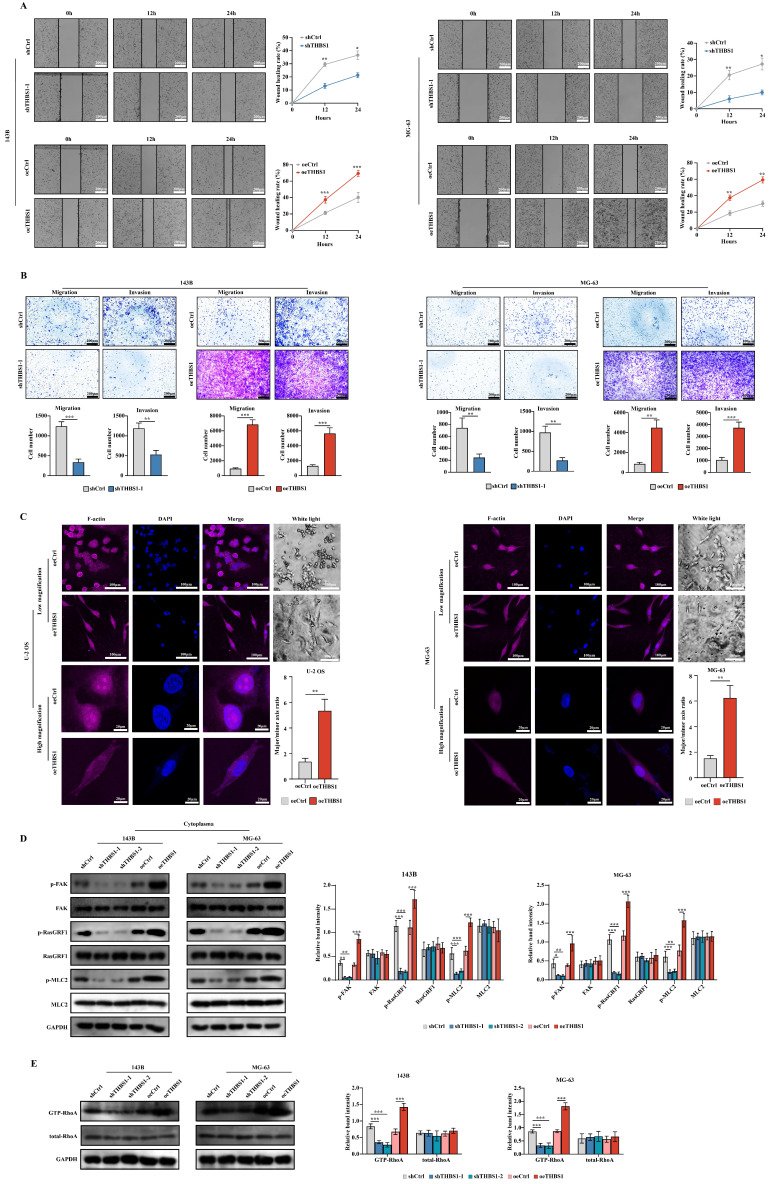
** The role and mechanism of THBS1 in osteosarcoma cell dedifferentiation and tumor malignancy.** (A) Wound-healing assay results of THBS1 knockdown and overexpression cells are shown. (B) Transwell migration and invasion assay results of THBS1 knockdown and overexpression cells are shown. (C) Cell morphological changes after expression of THBS1 in different osteosarcoma cell lines are shown. Cells were stained for F-actin (magenta), DAPI (blue). (D) The expression of p-FAK, FAK, p-RasGRF1, RasGRF1, p-MLC2 and MLC2 in THBS1 knockdown and overexpression cells was determined. (E) The expression of GTP-RhoA and total RhoA in each group was shown. ***P < 0.001, **P < 0.01, *P < 0.05.

Some research suggests the relationship between cell dedifferentiation and tumor metastasis^19^, ^21^, ^22^. In this section, we aimed to investigate whether THBS1 promotes osteosarcoma cell migration and invasion in ivtro. The wound-healing assay using 143B and MG-63 cells suggested that THBS1 affects cell migration; knockdown of THBS1 reduced cell migration, while overexpression of THBS1 increased cell migration (Figure 3A). Transwell migration and invasion assays also showed that knocking down THBS1 reduced cell migration and invasion, while overexpressing of THBS1 increased cell migration and invasion (Figure 3B). We found a significant change in cell morphology after THBS1 overexpression, which was demonstrated by F-actin staining (Figure 3C). These results suggest a cytoskeleton remodeling exist in the cells with THBS1 expression. Therefore, combining enrichment results from mRNA-seq, we assessed cell adhesion and cytoskeleton-related factors and found that the expression of p-FAK, p-RasGRF1, p-MLC2 and GTP-RhoA was positively correlated with the expression of THBS1 (Figure 3D-E).

### THBS1 promotes dedifferentiation via ITGA1 and ITGA6 in the early phase

**Figure 4:**
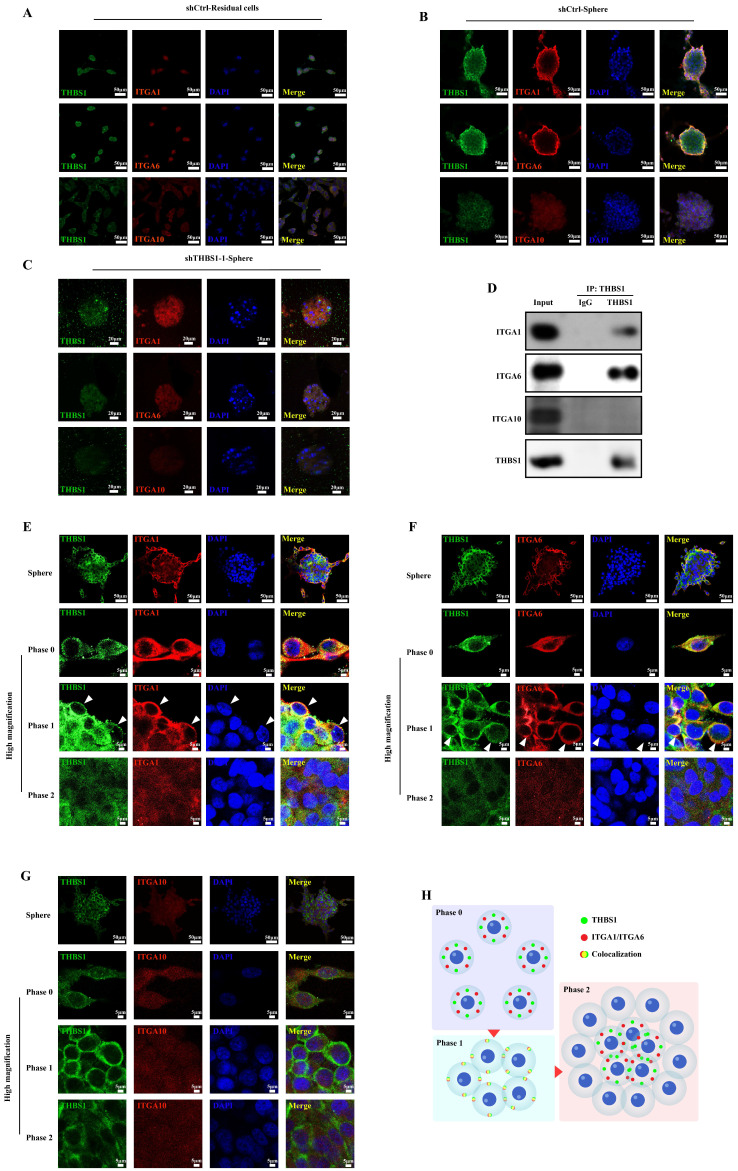
** The role and timing of THBS1 and ITGAs in osteosarcoma cell dedifferentiation.** (A-C) Immunocytochemistry was utilized to evaluate THBS1 (green), ITGA1 (red), ITGA6 (red) and ITGA10 (red) expression in residual cells and sarcospheres. Residual cells and sarcospheres were counterstained with DAPI (blue). (D) Co-IP experiments were performed to determine the interaction between THBS1 and ITGAs. (E-G) The colocalization of THBS1 and ITGAs in different phases of osteosarcoma cell dedifferentiation was assessed. (H) Scheme of the colocalization of THBS1 and ITGAs in different phases of osteosarcoma cell dedifferentiation.

The results of the immunocytochemistry assay demonstrated that there was no obvious colocalization phenomenon between THBS1 and ITGAs in residual cells, but colocalization of THBS1 with ITGA1/ITGA6 was observed in sarcospheres, suggesting that there may be interactions between THBS1 and ITGA1, and between THBS1 and ITGA6 in sarcospheres (Figure 4A-B). In addition, during the sphere formation of osteosarcoma cells, the signal intensity of ITGA1 and ITGA6 on the cell membrane surface at the outer edge of the sphere was higher than that inside the sphere, and colocalization with THBS1 also occurred in cells at the outer edge of the sphere. In contrast, the ITGA10 signal did not show similar changes and did not colocalize with THBS1 (Figure 4B). As expected, the colocalization between THBS1 and ITGA1/ITGA6 disappeared after knocking down THBS1 (Figure 4C). Co-IP experiments confirmed the interaction between THBS1 and ITGA1/ITGA6 in the dedifferentiation of osteosarcoma cells (Figure 4D). We defined phase 0 cells as cells that had not yet formed spheres, phase 1 cells as cells that had just participated in sphere formation and were at the outer edge of the sphere, and phase 2 cells as cells that had already participated in sphere formation and were inside the sphere. We observed that the colocalization of THBS1 and ITGA1, or THBS1 and ITGA6 only occurred in phase 1 (Figure 4E-G, white arrows indicate the typical cells in phase 1). These results suggest that THBS1 promotes dedifferentiation via ITGA1 and ITGA6 in the early phase, but this promotion may no longer be functional in the mature sarcosphers (Figure 4H).

### THBS1 and ITGAs do not play a role in promoting dedifferentiation inside mature sarcospheres

**Figure 5:**
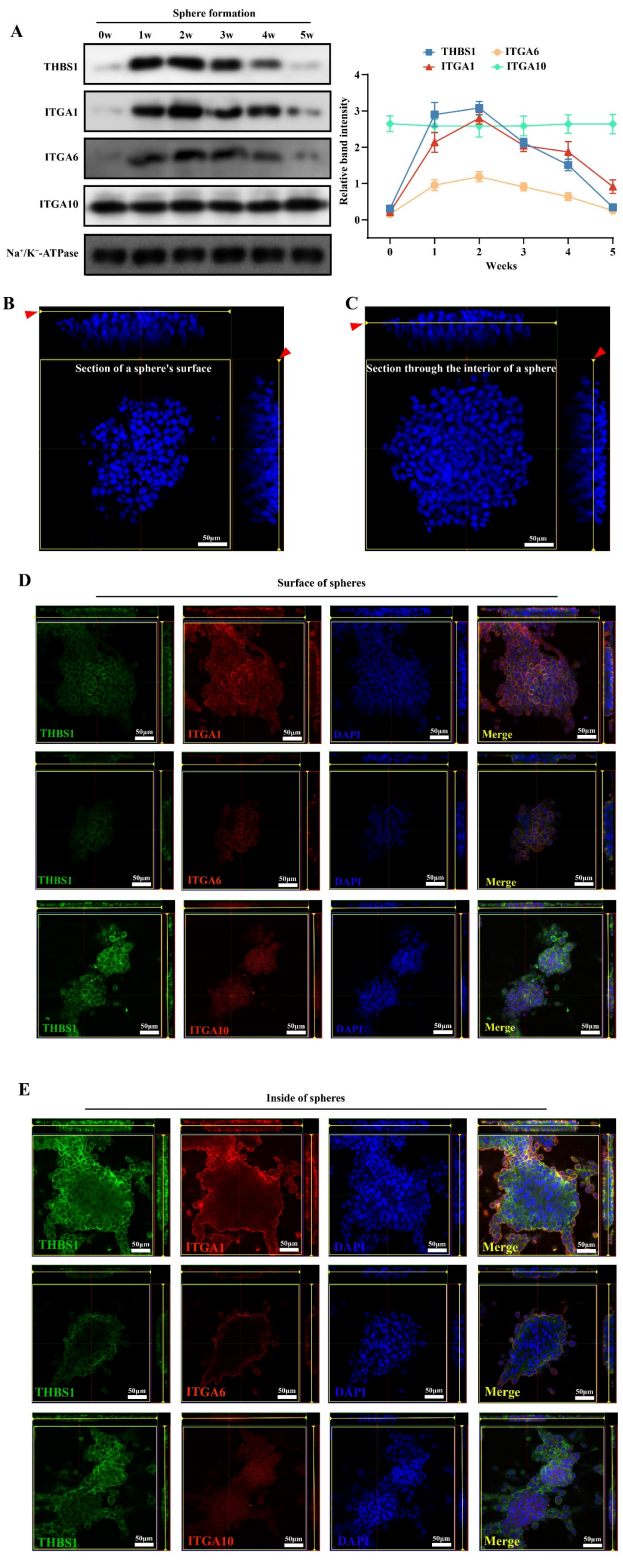
** The expression of THBS1 and ITGAs in mature sarcospheres.** (A) Expression changes in THBS1 and ITGAs on the cell membrane during different phases of osteosarcoma cell dedifferentiation are shown. A schematic diagram of obtaining immunofluorescence images of cells on the surface (B) or inside the sphere (C). The red arrows indicate the position of the sphere where the image is located. (D) The colocalization of THBS1 and ITGAs on the surface of mature sarcospheres was assessed. (E) The colocalization of THBS1 and ITGAs inside the mature spheres was assessed. Sarcospheres were stained for THBS1 (green), ITGA1 (red), ITGA6 (red), ITGA10 (red) and DAPI (blue).

To determine the specific stage at which THBS1 primarily influences the dedifferentiation of osteosarcoma cells, we analyzed the expression levels of THBS1 and ITGAs in osteosarcoma cells across various dedifferentiation time points. We found that the expression of THBS1, ITGA1, and ITGA6 in the cell membrane was relatively high from week 1 to 2 in the sphere formation process, indicating that these molecules were highly expressed in the early phase of dedifferentiation, but we also observed lower expression of THBS1, ITGA1 and ITGA6 in later phase of dedifferentiation (Figure 5A). To explain why the expression of THBS1, ITGA1, and ITGA6 in the cell membrane decreased in the late phase of sphere formation, we also assessed the expression pattern on the surface and inside of mature spheres that were subjected to a sphere formation assay for 4 weeks (Figure 5B-C) and found that THBS1, ITGA1, and ITGA6 had a strong signal only on the surface of mature spheres (Figure 5D-E).

### Knocking down of THBS1 inhibits pulmonary metastasis *in vivo*

**Figure 6:**
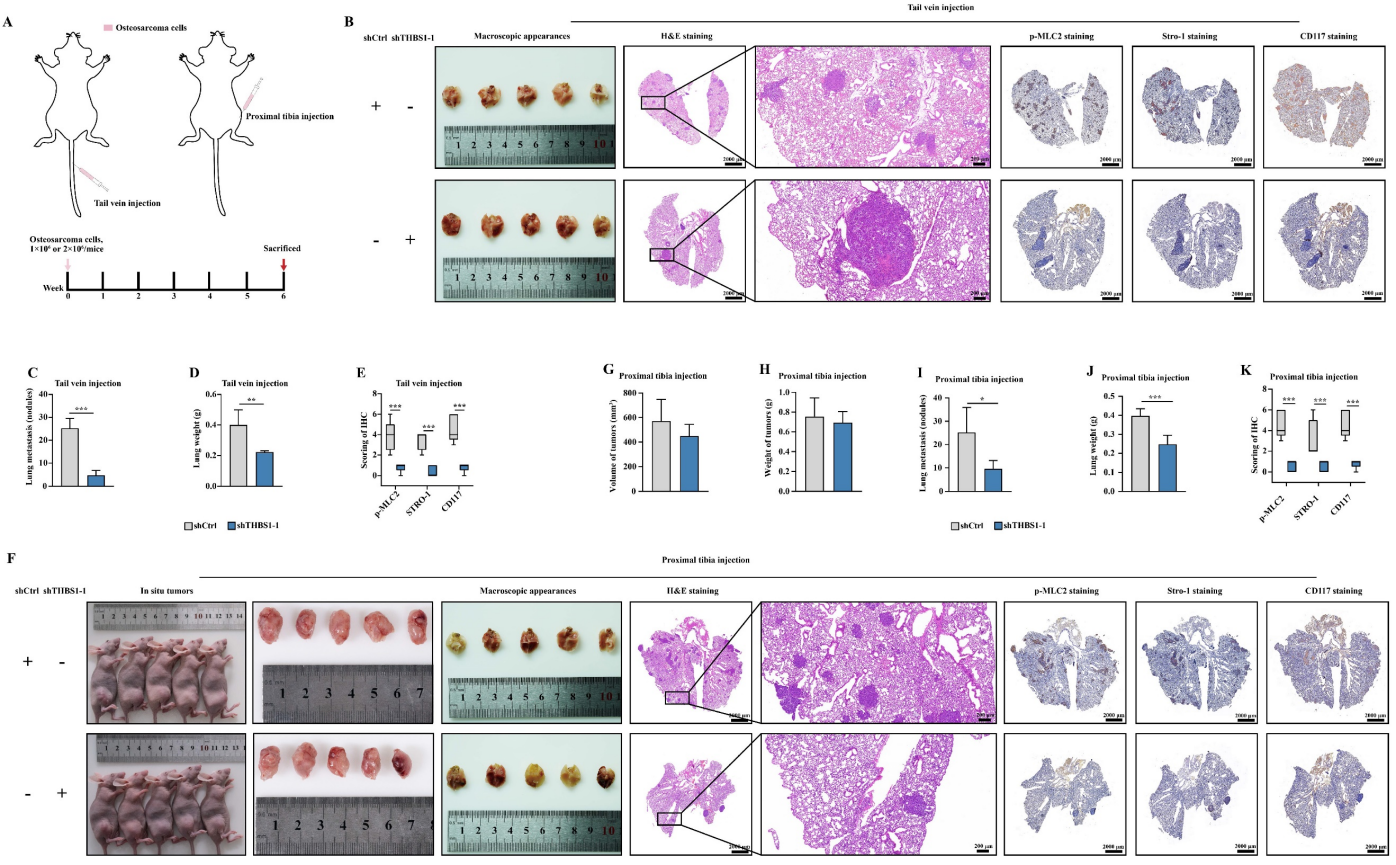
** Knocking down of THBS1 inhibits pulmonary metastasis *in vivo***. (A) The scheme for establishing pulmonary metastasis model in nude mice by tail vein injection or proximal tibia injection are shown. (B) A nude mouse pulmonary metastasis model was established by injecting tumor cells into the tail vein, showing the general morphology of the lungs, H&E staining, p-MLC2 staining, Stro-1 staining and CD117 staining. (C) The number of metastatic nodules was evaluated and analyzed in osteosarcoma cells transfected with lenti-shTHBS1 and the negative control group. (D) The weights of the lungs in shCtrl and shTHBS1-1 groups were determined. (E) Analysis of expression changes of p-MLC2, Stro-1 and CD117 in metastatic nodules following THBS1 knockdown. (F) A nude mouse pulmonary metastasis model was established by injecting tumor cells into the proximal tibia, showing the *in situ* tumors, general morphology of the lungs, H&E staining, p-MLC2 staining, Stro-1 staining and CD117 staining. (G-J) The volume and weight of *in situ* tumors, as well as the number of metastatic nodules and lung weights, were determined in both the shCtrl and shTHBS1-1 groups. (K) Analysis of expression changes of p-MLC2, Stro-1 and CD117 in metastatic nodules. ***P < 0.001, **P < 0.01, *P < 0.05.

To further clarify the ability of THBS1 to promote osteosarcoma metastasis, we first injected tumor cells through the tail vein to establish a nude mouse pulmonary metastasis model (Figure 6A). And, in order to simulate the clinical reality of tumor development more closely, we also established an osteosarcoma pulmonary metastasis model by injecting tumor cells into the proximal tibia of nude mice (Figure 6A). The results of the tail vein injection groups indicated that the knockdown of THBS1 reduced pulmonary metastasis in osteosarcoma (Figure 6B-D). Additionally, it decreased the expression levels of p-MLC2, Stro-1, and CD117 in the metastatic nodules (Figure 6B, 6E). The lung weights in subjects with THBS1 knockdown were significantly reduced, primarily due to the decrease in metastatic nodules (Figure 6D). Similar results can be seen in the proximal tibia injection groups (Figure 6F-K).

### THBS1 promotes osteosarcoma pulmonary metastasis via ITGA1 and ITGA6 *in vivo*

**Figure 7:**
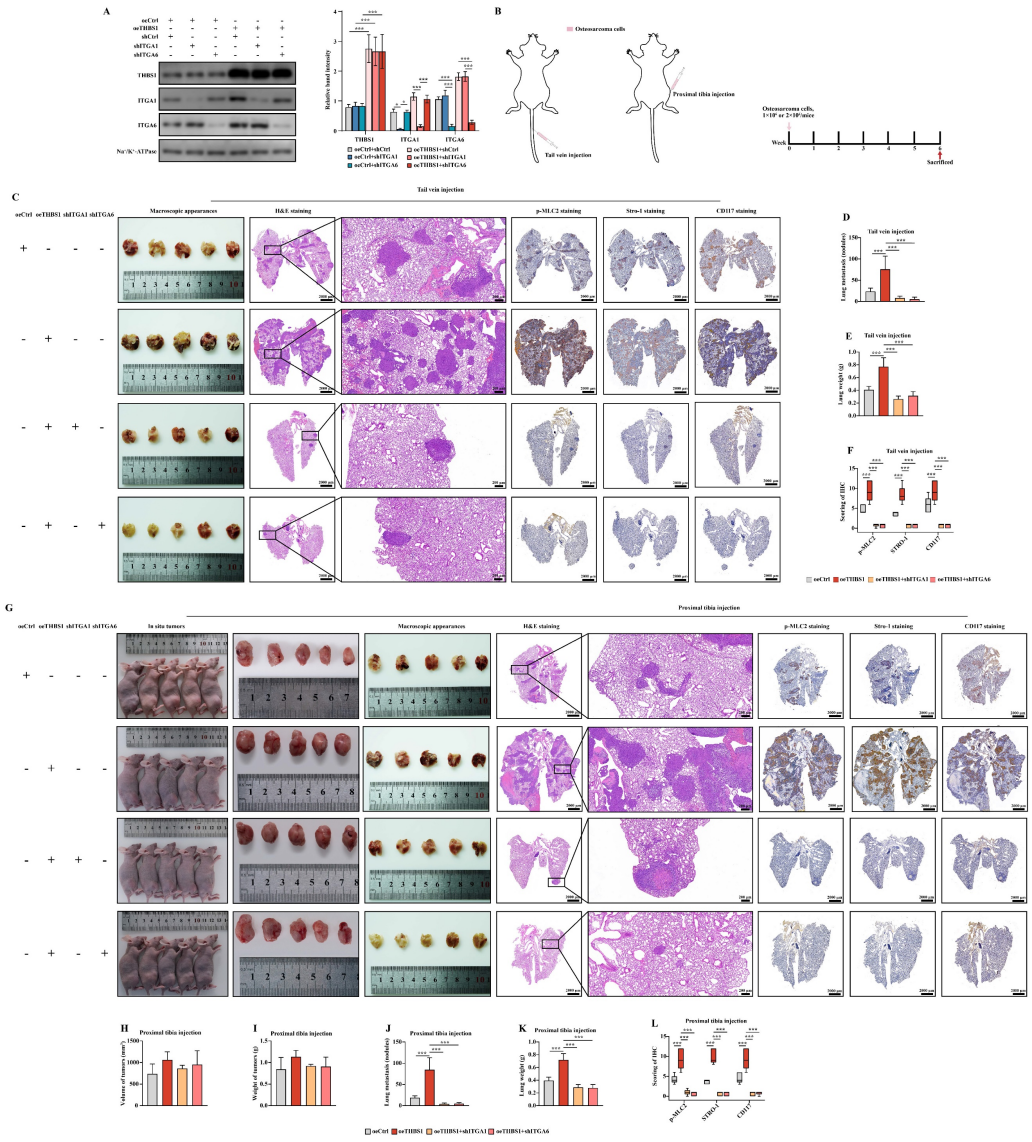
** THBS1 promotes pulmonary metastasis via ITGA1 and ITGA6 *in vivo***. (A) Western blotting was employed to show the knockdown of ITGA1 or ITGA6 in cells that overexpress THBS1. (B) The scheme for establishing pulmonary metastasis model in nude mice by tail vein injection or proximal tibia injection are shown. (C) The general morphology of the lungs, H&E staining, p-MLC2 staining, Stro-1 staining and CD117 staining of tail vein injection groups are shown. (D-E) The number of tumor nodules and the weights of the tail vein injection groups were evaluated and analyzed. (F) Analysis of the effects of THBS1 overexpression and ITGA1/ITGA6 knockdown on the expression of p-MLC2, Stro-1 and CD117 in metastatic nodules of tail vein injection groups. (G) The *in situ* tumors, general morphology of the lungs, H&E staining, p-MLC2 staining, Stro-1 staining and CD117 staining of proximal tibia injection groups are shown. (H-K) The volume and weight of *in situ* tumors, and the number of metastatic nodules and the weights of the proximal tibia injection groups were evaluated and analyzed. (L) Analysis of the effects of THBS1 overexpression and ITGA1/ITGA6 knockdown on the expression of p-MLC2, Stro-1 and CD117 in metastatic nodules of proximal tibia injection groups. ***P < 0.001, *P < 0.05.

The results of the western blotting indicated that the expression of ITGA1 and ITGA6 was knocked down in cells overexpressing THBS1 (Figure 7A). We established pulmonary metastasis model in nude mice again by tail vein injection or proximal tibia injection (Figure 7B). The results of tail vein injection groups showed that overexpression of THBS1 promoted osteosarcoma metastasis and then increased the lung wights (Figure 7C-E). Additionally, THBS1 enhanced the expression of p-MLC2, Stro-1, and CD117 *in vivo*, indicating its role in facilitating cytoskeleton remodeling and the dedifferentiation of osteosarcoma cells (Figure 7C, 7F). However, the role of THBS1 in promoting osteosarcoma pulmonary metastasis could be reversed by ITGA1 or ITGA6 knockdown (Figure 7C-F). Similar outcomes were observed in the groups receiving proximal tibia injections (Figure 7G-L).

### THBS1 promotes dedifferentiation and the cytoskeletal remodeling of primary osteosarcoma cells

**Figure 8:**
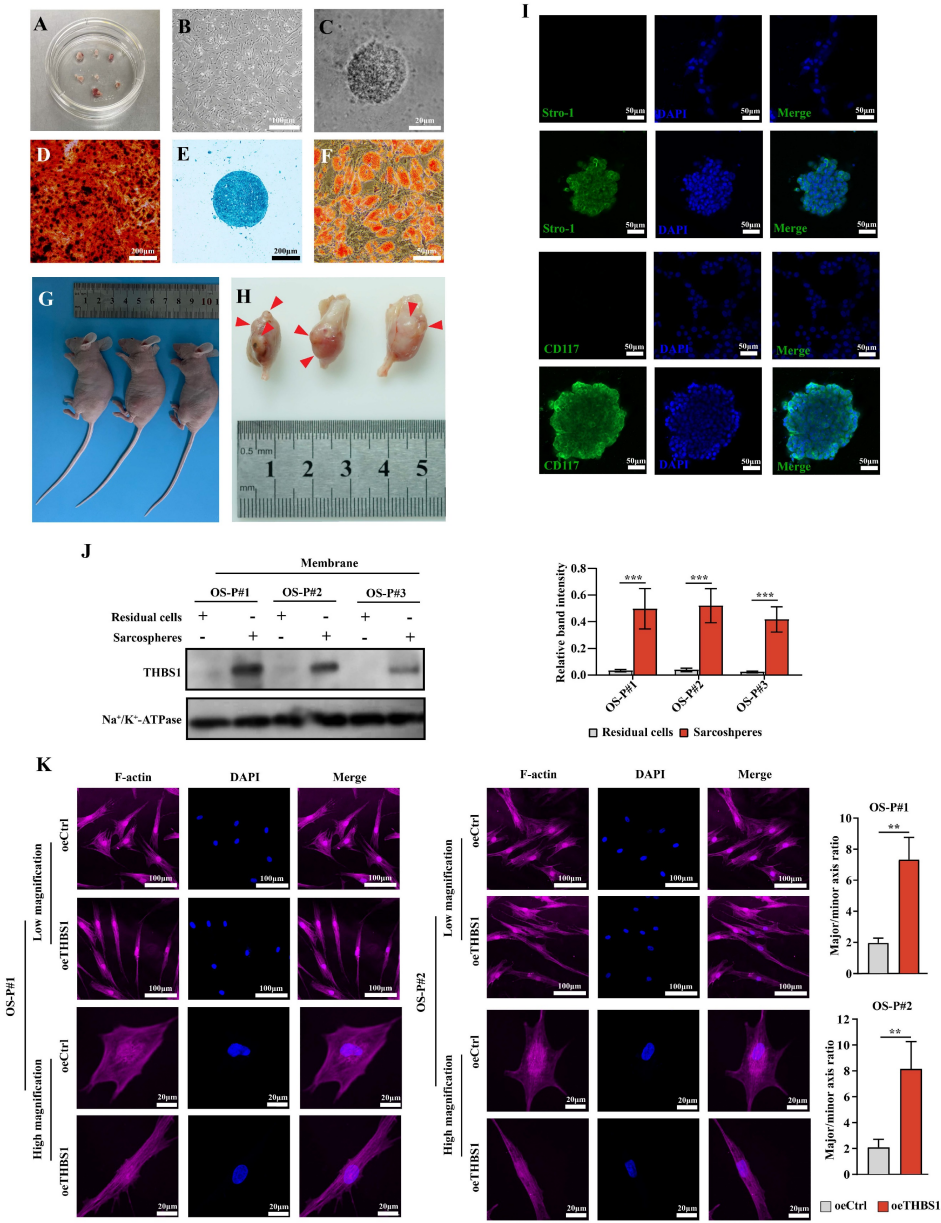
** The function of THBS1 in the dedifferentiation of primary osteosarcoma cells.** (A) Osteosarcoma primary cells were isolated from osteosarcoma tissues. (B) Osteosarcoma primary cell culture was performed. (C) Sphere formation of primary osteosarcoma cells was assessed. (D-F) A spheroid cell multidirectional differentiation experiment, including osteogenic, chondrogenic and adipogenic experiments, was performed. (G-H) A tumor formation experiment was performed in nude mice by injecting spheroid cells into the proximal tibia. (I) Immunocytochemistry was used to evaluate Stro-1 (green) and CD117 (green) expression in residual cells and sarcospheres. Residual cells and sarcospheres were counterstained with DAPI (blue). (J) The expression of THBS1 in formed spheres and residual cells in primary osteosarcoma cells derived from different patients was determined. (K) Changes in cell morphology after overexpression of THBS1 in primary osteosarcoma cells. Cells were stained for F-actin (magenta), DAPI (blue). ***P < 0.001, **P < 0.01.

Next, we investigated the role of THBS1 in the dedifferentiation of primary osteosarcoma cell. We isolated primary osteosarcoma cells from osteosarcoma surgical samples and cultured them *in vitro* (Figure 8A-B). Then, we performed the sphere formation assay to obtain dedifferentiated primary osteosarcoma cells (Figure 8C), and the ability of these cells to redifferentiate into osteoblasts, chondrocytes, and adipocytes was determined (Figure 8D-F). To confirm that the obtained cells were indeed tumor cells, we injected those cells into the proximal tibia of nude mice, and the results showed that the dedifferentiated primary osteosarcoma cells could form tumors in the tibia of nude mice (Figure 8G-H, red arrows indicate the formed tumors). The spheroid cells also had stem cell-like characteristics, showing that the spheroid cells were enriched in the expression of CD117 and Stro-1 when compared to the residual cells (Figure 8I). We also found that in the primary cells derived from 3 osteosarcoma patients, THBS1 was more highly expressed in sarcospheres relative to that in residual cells (Figure 8J). In addition, similar to osteosarcoma cell lines, which was demonstrated by F-actin staining, the overexpression of THBS1 in primary osteosarcoma cells promoted cytoskeletal remodeling (Figure 8K).

## Discussion

Osteosarcoma is the most common primary bone tumor, and it is highly malignant, grows rapidly, and is painful. Targeting the dedifferentiation process could yield new approaches to solve certain problems, such as pulmonary metastasis and unfavorable prognosis.

To begin our research, we need to obtain dedifferentiated osteosarcoma cells. Mature differentiated cells in tumor tissue can serve as a reserve of stem cells during the development of tumors. These cells acquire stem cell properties under the accumulation of endogenous genetic mutations and the stimulation of exogenous environmental signals and play a role in tumor initiation and development^27^. When dedifferentiated tumor cells are cultured *in vitro*, they are typically characterized by a spherical shape suspended in the medium. Gibbs' research showed that osteosarcoma cells have the ability of forming sarcospheres, and some osteosarcoma cells express mesenchymal stem cell markers, such as Stro-1, CD44, and CD105. Compared with adherent cells, osteosarcoma spheres significantly highly express the embryonic pluripotent stem cell marker molecules Oct4 and Nanog^28^. Murase *et al.* demonstrated that sarcospheres can be cultured from osteosarcoma cell lines such as the OS2000, KIKU, NY, Huo9, HOS, U-2 OS, and Saos2. The formed sarcospheres have stem cell characteristics and have tumor-forming ability *in vivo* in the NOD/SCID mice^29^. In addition, after long-term treatment of MG-63 cells with 3-aminobenzamide (3-AB), a new cell line, 3AB-OS, with stem cell characteristics was obtained. It indicated 3AB-OS underwent dedifferentiation and these cells had a stronger self-renewal ability and tumorigenic ability and highly expressed the stemness genes CD133, Oct3/4, Nanog, and ABCG2^30^, ^31^. In this study, we successfully obtained dedifferentiated osteosarcoma cells through sphere formation experiments, which provides a material basis for subsequent research.

Exploring how mature differentiated osteosarcoma cells dedifferentiate into progenitor cells with stem cell characteristics and why these cells are prone to lung metastasis is important. Our results demonstrated that THBS1 and ITGAs are the key molecules in osteosarcoma cell dedifferentiation. Previously, THBS1 was reported to be an inhibitor of angiogenesis and tumor progression^32^-^34^. However, recent studies have reached the contradictory conclusion that THBS1 promotes tumor migration, invasion, and distant metastasis in breast cancer, thyroid cancer, colon cancer, prostate cancer, and melanoma^35^-^40^. Lawler reported that fewer osteosarcomas occurred in p53-deficient mice that lack THBS1 than in those that express THBS1^41^. In addition, the knockout of THBS1 in an animal model of breast cancer led to growth of the primary tumor but a decrease in the number of metastases^39^.

Integrins interact with many ligands, including ECM proteins, VCAM-1, and ADAM family members. THBS1 is a multifunctional protein that interacts with several cell surface receptors, including integrins, such as α3β1^42^-^45^, α4β1^46^, ^47^, α6β1^48^, ^49^, α9β1^50^, ανβ1^51^, ανβ3^52^. These integrins regulate THBS1 to promote the adhesion and migration of tumor cells, vascular cells, and T cells^43^, ^44^, ^47^. The RGD sequence in the calcium-binding repeats of THBS1 is recognized by ανβ3^53^. In brief, THBS1 is a cell adhesion-related factor and a multifunctional receptor that interacts with ECM proteins through integrins. It aligns with our prior analysis, which employed GO, KEGG, and PPI to identify factors associated with the dedifferentiation of osteosarcoma cells. Our results also demonstrated that ITGA1 and ITGA6, two subunits of integrin, play a role in mediating extracellular signals in cells and that THBS1 promotes the dedifferentiation and metastasis of osteosarcoma cells.

Some signaling pathways have been reported to be involved in cell migration that is regulated by THBS1, including the ERK, P38MAPK, and FAK pathways^40^, ^54^-^57^. Hu *et al.* reported that THBS1 promotes pulmonary metastasis of osteosarcoma through FAK dependent pathway^58^. Our results revealed that THBS1 promotes cytoskeleton remodeling and pulmonary metastasis in osteosarcoma, and FAK play a role in this process. During the invasion and metastasis of tumors, tumor cells at the primary site invade local capillaries or lymphatic vessels through adhesion and invasion, spread to distant locations, are absorbed by capillaries or lymphatic vessels at a specific site, and then undergo subsequent settling and malignant proliferation around the blood vessels in secondary sites, eventually leading to tumor metastasis^59^-^61^. Tumor cells undergo EMT to migrate and invade first before distant metastasis^62^-^64^. During the migration and invasion of tumor cells, F-actin is reorganized at the front of the tumor cell to promote the formation of protrusions on the cell membrane^65^, ^66^, and the receptors on the protrusions on the cell membrane interact with extracellular matrix substrates to combine and recruit relevant molecules to form cell-ECM adhesion^67^. After forming adhesions, protrusions secrete proteases into the ECM to dissolve the ECM^68^-^70^. Then, the intracellular actin cytoskeleton contracts with the stress provided by the adhesions, thereby pulling the cell forward^71^. Finally, the adhesions of the cell dissolve and move forward with the cell body. In summary, cytoskeletal remodeling and cell adhesion play an important role in the EMT. However, sarcomas, like osteosarcoma, are not epithelial in origin and cannot become more “mesenchymal”^72^-^74^, so Lamhamedi-Cherradi *et al.* prefer to describe the EMT-like epigenetic effect as dedifferentiation^22^, which describes rejuvenated cell fate as cells shift up the Waddington epigenetic differentiation landscape^75^.

The functions of FAK and RasGRF1 are probably closely related and they likely play a role in the process of dedifferentiation. Huang *et al.* reported that decreased expression of adhesion and cytoskeleton organization-related molecules, such as FAK, RsaGRF1, RhoA, and ROCK, was observed with the inhibition of EMT in 5-8F-DLC-1 cells^76^. Research in 2020 demonstrated that FAK and RsaGRF1 were enriched in focal adhesion and expressed differentially when Fat mass- and obesity-associated (FTO) gene was overexpressed^77^. Baldelli *et al.* reported increased activation of FAK Y576/577 and higher expression of RasGRF1 in KRASm/WT^-^ cells than in KRASm/WT^+^ and WT cells^78^. In addition, researchers have indicated that RasGRF1, RasGFR2, and RasGRP are associated with the activation of FAK^79^. RasGRF1 is a Ras-selective guanyl exchange factor (GEF)^80^. GEFs activate GTPase by stimulating the release of guanosine diphosphate (GDP) to allow the binding of guanosine triphosphate (GTP)^81^, ^82^. Rho GTPase plays a key regulatory role in cell morphology, motility, proliferation, and apoptosis through cytoskeleton remodeling and dynamics^83^. ROCKs are activated when GTP-bound RhoA/C binds to the Rho-binding domain (RBD), which is located in the coiled-coil region of ROCKs^84^-^86^. Subsequently, ROCKs mediate the phosphorylation of downstream target proteins, such as MLC, LIMK1/2, MYPT1, and adducin, to promote actin contraction^87^-^93^. Our results showed cytoskeleton remodeling after overexpression of THBS1, and the expression of p-FAK, p-RasGRF1, and p-MLC2 was positively correlated with the expression of THBS1.

Osteosarcoma is a malignant bone tumor commonly found in young individuals. The survival prognosis for patients is poor due to their susceptibility to early pulmonary metastasis. Current treatment strategies can effectively eliminate most tumor cells; however, there remains a risk of recurrence and lung metastasis. Our research focuses on a specific subset of cells exhibiting high metastatic potential, particularly those with stem cell-like characteristics that are resistant to conventional drugs. We aim to promote the redifferentiation of these cells from a poorly differentiated state into highly differentiated tumor cells that are more susceptible to chemotherapy, while also preventing other tumor cells from undergoing dedifferentiation. Our research has identified the key molecule THBS1, along with its downstream targets ITGA1 and ITGA6, which play a role in the dedifferentiation of osteosarcoma cells. In the future, we aim to further explore the binding sites of THBS1 with ITGA1 and ITGA6. We also plan to develop competitive binding peptides to disrupt the signaling pathway of THBS1, which promotes the dedifferentiation of osteosarcoma. This research may contribute to the development of drugs that inhibit the dedifferentiation of osteosarcoma cells.

However, this study is still limited because we did not fully determine how THBS1 interacts with ITGA1/ITGA6 and the domains in which they interact. Once we identify the structural region where the interaction occurs, short peptide drugs that competitively bind to this domain may block the function of THBS1 in promoting the dedifferentiation of osteosarcoma cells.

**Figure 9:**
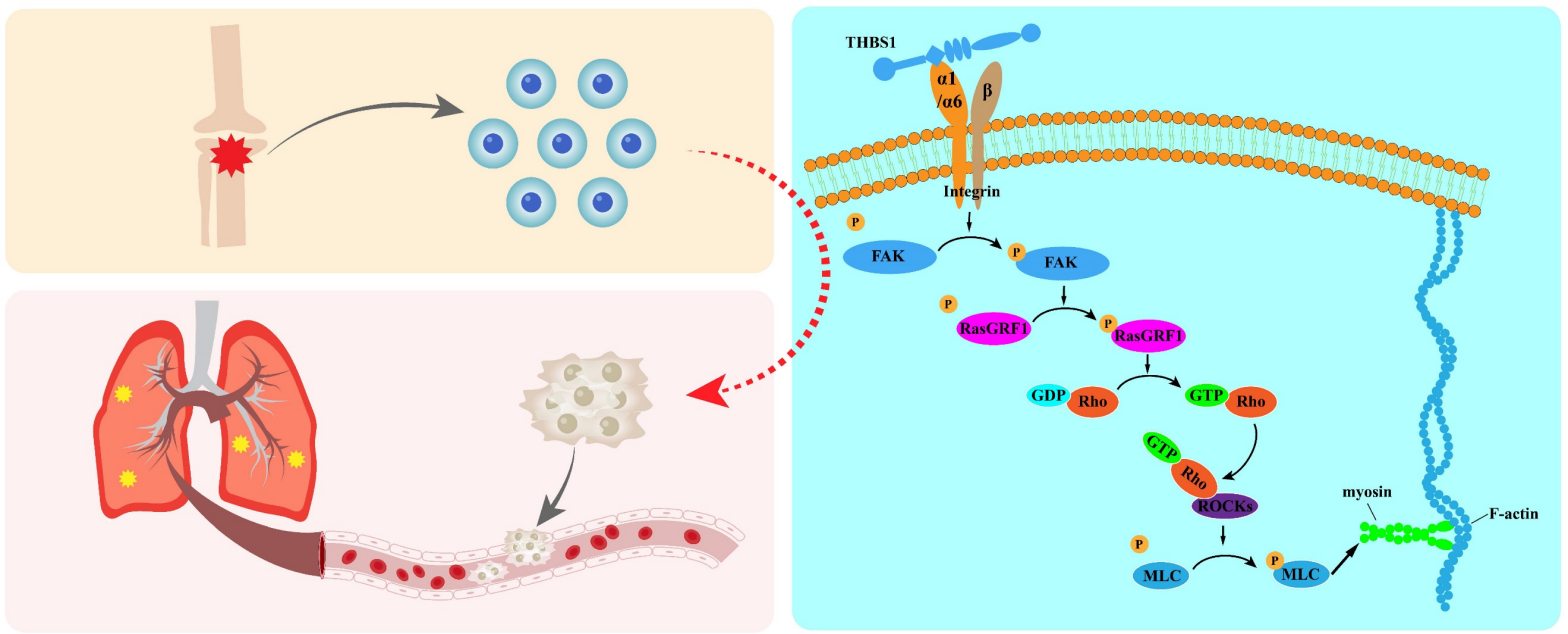
Graphical abstract.

In summary, our results indicated that THBS1 promotes cell dedifferentiation and metastasis by promoting cytoskeletal remodeling, and that ITGA1 and ITGA6 play an important role in mediating extracellular to intracellular signals (Figure 9); this mediating effect mainly occurred in the early phase of cell dedifferentiation. Our finding identified key molecules that promote the dedifferentiation of osteosarcoma cells, providing valuable insights for developing drugs to inhibit this process and reduce the risk of pulmonary metastasis in osteosarcoma.

## Abbreviations

THBS1: thrombospondin 1
ITGA: integrin subunit alpha
FAK: focal adhesion kinase
RasGRF1: ras protein-specific guanine nucleotide releasing factor 1
GEF: guanyl exchange factor
RhoA: ras homolog family member A
MLC2: myosin light chain 2
ROCK: rho-associated coiled-coil kinase
DEGs: differentially expressed genes
GO: gene ontology
KEGG: Kyoto encyclopedia of genes and genomes
PPI: protein-protein interaction
ITS-X: insulin-transferrin-selenium-ethanolamine
IHC: immunohistochemistry
ICC: immunocytochemistry
NGS: normal goat serum
Co-IP: co-immunoprecipitation
FBS: fetal bovine serum
BSA: bovine serum albumin
GDP: guanosine diphosphate
GTP: guanosine triphosphate
